# Binge drinking associated with mean temperature: a cross-sectional study among Mexican adults living in cities

**DOI:** 10.1186/s12992-024-01033-z

**Published:** 2024-04-12

**Authors:** Martha Carnalla, Nancy López-Olmedo, Yenisei Ramírez-Toscano, Luz Mery Cárdenas-Cárdenas, Francisco Canto-Osorio, Herney Rengifo-Reina, David Barrera-Núñez, Josúe Alai Quiroz-Reyes, M. Arantxa Colchero, Tonatiuh Barrientos-Gutiérrez

**Affiliations:** 1 Centro de Investigación en Salud Poblacional, Instituto Nacional de Salud Pública, Av. Universidad 655, Santa María Ahuacatitlán, 62100 Cuernavaca, Morelos, México; 2 Centro de Investigación en Sistemas de Salud, Instituto Nacional de Salud Pública, Av. Universidad 655, Santa María Ahuacatitlán, 62100 Cuernavaca, Morelos, México

**Keywords:** Binge drinking, Temperature, Alcohol consumption

## Abstract

**Background:**

The association between environmental temperature and alcohol consumption has not been widely explored despite the potential that increasing temperatures could promote the consumption of alcoholic beverages and the alcohol-related burden of disease. We aimed to explore the association between temperature and binge drinking in Mexican adults from urban cities, overall, and by alcoholic beverage type.

**Methods:**

Data on 10,552 adults ≥ 18 years was obtained from the 2016 National Survey on Drug, Alcohol, and Tobacco Consumption. The mean annual temperature at the municipality was obtained from the Mexican National Weather Service using monthly temperatures from 2015 to 2016. We analyzed binge drinking for all alcoholic beverages in the last year and by type of alcohol as beer, liquor, wine, and coolers. Associations between mean temperature over the past year and binge drinking over the past year among current drinkers were estimated using multilevel Poisson models with robust standard errors adjusted for age, sex, education level, marital status, and household socioeconomic status, with a fixed effect by region.

**Results:**

We observed a non-significant increase in the prevalence of binge drinking for every difference of 1 °C between municipalities of the same region. By alcohol type, a 1 °C increase in mean annual temperature across municipalities of the same region increased the prevalence of beer binge drinking in the past year by 0.9% (PR = 1.009, 95%CI 1.005, 1.013) among beer consumers and the prevalence of coolers’ binge drinking by 3.0% (PR = 1.030, 95%CI 1.003, 1.057) in coolers consumers. We observed non-significant results for liquor binge drinking (PR = 1.047, 95%CI 0.994, 1.102) and wine binge drinking (PR = 1.047, 95% 0.944, 1.161).

**Conclusion:**

People living in municipalities with higher temperatures reported a higher beer binge drinking in Mexican cities. This could account for 196,000 cases of beer binge drinking in 2016. The context of each country needs to be considered when generalizing these findings, and they need to be further explored with longitudinal data as there might be implications for climate change. If our findings are confirmed given the forecasted rising temperatures, we could expect an increase in binge drinking and therefore, in the alcohol burden of disease.

**Supplementary Information:**

The online version contains supplementary material available at 10.1186/s12992-024-01033-z.

## Introduction

Alcohol consumption increases the risks of more than 200 health problems, including infectious diseases, non-communicable diseases, mental disorders, violence, and injuries [1]. These risks are even higher with patterns of binge drinking (i.e., heavy-episodic alcohol consumption), defined as consuming 60 g of pure alcohol per occasion [2]. Individuals who engage in binge drinking, while not always meeting the diagnostic criteria of alcohol use disorder, exhibit a substantially elevated risk of transitioning to such a disorder in the future [3]. Individual conditions such as age, sex, and education level are associated with heavy alcohol consumption [4], yet contextual factors also play a role in binge drinking. Prior studies have analyzed the link between binge drinking and contextual level determinants, such as the density of alcohol outlets, prices [5], and social settings [6]. However, environmental determinants of alcohol consumption, such as temperature, remain understudied.

Climate change refers to persistent changes in the mean or the variability of key climate properties, such as temperature and precipitation [7]. Since 1982, an annual 0.2 °C rise per decade in temperature globally has been observed [8], a clear signal that climate change is rapidly occurring on our planet. As temperatures rise, behaviors influenced by temperature are also expected to increase. For instance, it has been observed lower consumption of fruit and vegetables but higher consumption of sugar-sweetened beverages at higher temperatures [9]. Alcohol consumption has a seasonal pattern [10]. For example, alcohol consumption increases from April to July and decreases from July to November in Sweden [11], and beer sales have the same pattern in Estonia [12]. This suggests that hotter temperatures could influence alcohol consumption, yet, very few studies have generated evidence about the potential impact of rising temperatures on alcohol behaviors, such as binge drinking.

Evidence about a potential link between binge drinking and temperature has been mixed to date. A study in Scotland found that binge drinking was higher during summer and autumn [13]. In contrast, a study using US data found a negative association between temperature and binge drinking [14]. Although, the associations found in these studies can be heavily confounded by the sociocultural characteristics; the ecological design does not allow for extrapolation of the results at the individual level, as previously discussed by other authors [15, 16]. Contextual factors, such as national or subnational (i.e., regions or states) preferences of alcoholic drinks according to temperature could explain the lack of a homogeneous finding, and studies originating from different contexts could help understand this complex relationship.

Contextual factors influence the type and quantity of alcohol consumed across and within countries [17]. In Mexico in 2016, the national prevalence of binge drinking in the last month was 19.8% with heterogeneity across regions; the lowest prevalence was 16.8% in the South and the highest was 25% in North Central [18]. Also, by 2016, 77% of the alcohol consumed in Mexico was beer [2], a beverage that is culturally considered refreshing and is more frequently consumed during the summer. Still, there is heterogeneity across country regions, with a higher beer consumption in the North and South regions [19], which have higher temperatures than the Center [20]. Assessing the relationship between binge drinking and temperature considering the type of alcohol and the potential heterogeneity across regions can inform policies that reduce the harmful use of alcohol in the era of climate change.

In Mexico, temperature in cities with more than 100,000 inhabitants has increased on average 0.1 °C per year since 2007 [9]. Binge drinking is more prevalent in urban and metropolitan areas [21]. Increasing temperatures could further promote the consumption of alcoholic beverages and increase the alcohol-related burden of disease. Taking advantage of the rich climate and the information on sociodemographic characteristics and alcohol consumption data at the individual level, we aimed to explore the cross-sectional association between temperature and binge drinking in Mexican cities, overall, and by alcohol type (beer, liquors, wine, and coolers). We hypothesized that higher temperatures will be associated with higher beer binge drinking considering that beer is the most prevalent alcoholic beverage consumed in Mexico.

## Methods

### Study design and population

We aimed to explore the association between temperature and binge drinking in Mexican adults from cities, overall, and by alcoholic beverage type. We used individual-level data from adults ≥ 18 years old (who legally can purchase alcohol in Mexico) who participated in the 2016 National Survey on Drug, Alcohol, and Tobacco Consumption (ENCODAT) in Mexico. We used the 2016 data because it is the latest year the survey was conducted. ENCODAT is a nationally representative survey with a probabilistic, multistage, stratified design with a response rate of 73.6%. An adult aged 18 to 65 and an adolescent aged 12 to 17 were randomly selected in each household. They answered a questionnaire on the use of different psychoactive substances, including alcohol. The survey was collected from June 1st to October 31st, 2016. Further details of the survey are presented elsewhere [22]. 

We included 11,722 current drinkers (defined as alcohol consumption in the last 12 months) aged 18 years and older living in urban areas as defined by the *Salud Urbana en América Latina* (SALURBAL) project. SALURBAL defines cities as urban clusters of municipalities with more than 100,000 inhabitants [23]. We excluded individuals without survey date (*n* = 2), living in places without station data on temperature (*n* = 589), with > 6 months missing value for annual temperature after imputation (*n* = 40), or with missing covariates (*n* = 57). This left a total of 11,034 participants that could be successfully linked to temperature measurements. These participants were in 257 municipalities with a median number of 127 participants (IQR 137) per municipality.

Exposure variables.

We used area-level temperature data from the weather stations provided by the Mexican National Weather Service on monthly temperatures from 2015 to 2016 [24]. Individual-level data from ENCODAT were linked to the municipality-level data from the National Weather Service using a unique municipality identifier. A municipality is defined as a political and administrative-territorial division of a state [25]. We built a database with the nearest stations to each municipality up to a 40 km geodesic distance estimated by latitude and longitude. We had 50% of weather stations with at least one missing month between May 2015 to October 2016. In these cases, we imputed temperature values corresponding to the same month of a previous or posterior year under the assumption of no extreme changes in temperature across years. For instance, if we identified a missing temperature value in May 2015, we imputed the value for May 2016. If the missing value was for October 2016, we imputed the monthly temperature for October 2015. Afterward, 8.0% of stations remained with at least one missing month.

We calculated the mean annual temperature with the closest station available for each municipality. We estimated the mean annual temperature defined as the average of the temperature across the month of the survey and the previous 11 months for each participant. All individuals in the same municipality who completed the survey in the same month were assigned the same annual mean temperature.

### Outcome variables

We estimated binge drinking in the last year in those who reported alcohol consumption in the past twelve months. Binge drinking was defined as consuming at least 5 and 4 standard alcoholic drinks in males and females, respectively, on at least one occasion in the last year. We categorized types of alcohol as beer, liquors (brandy, tequila, rum, whiskey), wine, and coolers. We defined individuals as current drinkers of such alcoholic beverages if they reported consuming them in the last year. We analyzed five dichotomous (yes/no) outcomes of alcohol consumption at the individual level: (**1**) binge drinking of any alcoholic beverage in the last year among current drinkers of any type of alcohol (*n* = 10,552, excluding *n* = 482 non-respondent), (**2**) beer binge drinking in the last year in beer current drinkers (*n* = 8,911, non-respondent *n* = 542), (**3**) liquor binge drinking in liquor current drinkers (*n* = 3,832, non-respondent *n* = 517), (**4**) wine binge drinking in the last year in wine current drinkers (*n* = 1,823, non-respondent *n* = 358), and (**5**) coolers binge drinking in the last year in coolers current drinkers (*n* = 1,348, non-respondent *n* = 241).

### Covariates

We considered the following self-reported covariates: age, sex assigned at birth, education, marital status, household socioeconomic status, and region of residence. Age was analyzed as a continuous variable in years; sex was measured as male or female; education was classified into none, elementary, middle, high school, or graduate; and marital status categories were single, cohabitating, separated/divorced, or widowed. Using all the household data from the ENCODAT survey, we created a household socioeconomic status index. We constructed a polychoric matrix using the following self-reported household variables: durable assets (car, computer, DVD player, microwave, internet, cable, telephone), kitchen, number of bulbs, and without overcrowding defined as 3 or fewer individuals by room. We then performed a principal component analysis, and selected the first factor (out of ten) with the highest eigenvalue (4.6) which explained 46.0% of the variability. We divided the index into quartiles; a higher quartile indicated a better household socioeconomic status [26]. We defined eight regions according to the state of residence as the original survey (ENCODAT): Central North (Coahuila, Chihuahua, Durango), North West (Baja California, Baja California Sur, Sonora, Sinaloa), North East (Nuevo León, Tamaulipas, San Luis Potosí), West (Zacatecas, Aguascalientes, Jalisco, Colima, Nayarit), Center (Puebla, Tlaxcala, Morelos, State of México, Hidalgo, Querétaro, Guanajuato), Mexico City, Central South (Veracruz, Oaxaca, Guerrero, Michoacán), and South (Yucatán, Quintana Roo, Campeche, Chiapas, Tabasco).

### Statistical analysis

We estimated the mean and standard deviation for continuous variables and frequency and percentages for categorical variables in current drinkers of any alcoholic beverages and by type of alcohol. We conducted an alcoholic beverage binge drinking type-specific analysis and the overall measure of binge drinking for temperature analyses.

To estimate the association between temperature and alcohol consumption, we fitted multilevel Poisson models for each outcome adjusted for age, sex, education level, marital status, and household socioeconomic status, with a fixed effect by region—to account for unobserved differences across regions (social, cultural, environmental)— and robust standard errors considering the clustering by the municipality. We used a Poisson model to estimate the prevalence ratio, given that it is a better alternative for cross-sectional analysis than logistic regression when the prevalence of the outcome is common (> 10%) [27]. The multilevel model accounts for the temperature as a contextual exposure, i.e. a group of individuals are exposed to the same temperature if they live in the same municipality [28]. Results are presented as prevalence ratios (PR) associated with a 1 °C increase in temperature. According to exploratory analyses of each type of alcoholic beverage, liquor, wine, and coolers binge drinking had a non-linear association with temperature (see Additional Figs. 1, 2, 3 and 4); thus, a cubic polynomial term was included in these models.

We performed two sensitivity analyses to evaluate differences in the estimator when more precise exposure measurements were used: (1) the sample restricted to individuals in locations with complete monthly temperature values for the period 2015–2016; (2) the sample further restricted to individuals in locations with climate stations within a 1 km ratio. We described the characteristics of each of these samples in Additional Table 1. All analyses were performed in Stata 17 (College Station, TX).

## Results

The mean annual temperature was 21.9 °C (min 12.3 °C, max 29.7 °C). Table 1 shows the characteristics of current drinkers overall and by type of alcohol. Among current drinkers, the mean age was 38 years, 51% were male, and most participants completed middle school, followed by high school education. Cohabitation was reported by 59% of the participants, and 41% were in the highest quarter of household socioeconomic status. These characteristics were similar across types of alcoholic beverages, yet liquor and wine drinkers had a higher education level and a higher household socioeconomic status than beer drinkers. Current cooler drinkers were younger and more often single than beer drinkers (Table 1).

**Table 1:** Characteristics of current drinkers overall and by type of alcoholic beverage in the last year

	Current drinkers	Beer drinkers	Liquor drinkers	Wine drinkers	Coolers drinkers
	n (%)	n (%)	n (%)	n (%)	n (%)
Prevalence		84.4	36.3	12.3	12.8
Age, years (mean ± SD)	37.5 ± 12.8	36.9 ± 12.6	35.8 ± 12.9	37.1 ± 13.1	32.1 ± 11.3
Sex					
Male	5,392 (51.1)	4,883 (54.8)	2,073 (54.1)	901 (49.4)	692 (51.3)
Female	5,160 (48.9)	4,028 (45.2)	1,759 (45.9)	922 (50.6)	656 (48.7)
Education					
None	549 (5.2)	472 (5.3)	119 (3.1)	46 (2.5)	24 (1.8)
Elementary	1,541 (14.6)	1,310 (14.7)	406 (10.6)	129 (7.1)	127 (9.4)
Middle-school	3,651 (34.6)	3,137 (35.2)	1,127 (29.4)	438 (24.0)	462 (34.3)
High-school	3,028 (28.7)	2,549 (28.6)	1,314 (34.3)	594 (32.6)	495 (36.7)
Graduate	1,783 (16.9)	1,435 (16.1)	862 (22.5)	616 (33.8)	239 (17.7)
Marital status					
Single	3,081 (29.2)	2,647 (29.7)	1,391 (36.3)	658 (36.1)	568 (42.1)
Cohabitating	6,257 (59.3)	5,275 (59.2)	2,058 (53.7)	975 (53.5)	638 (47.3)
Separated/Divorced	992 (9.4)	811 (9.1)	326 (8.5)	166 (9.1)	131 (9.7)
Widowed	222 (2.1)	169 (1.9)	57 (1.5)	24 (1.3)	12 (0.9)
Socioeconomic status					
Q1	1,414 (13.4)	1,230 (13.8)	360 (9.4)	120 (6.6)	148 (11.0)
Q2	1,889 (17.9)	1,613 (18.1)	517 (13.5)	191 (10.5)	191 (14.2)
Q3	2,912 (27.6)	2,495 (28.0)	985 (25.7)	396 (21.7)	357 (26.5)
Q4	4,337 (41.1)	3,564 (40.0)	1,970 (51.4)	1,116 (61.2)	651 (48.3)

SD: Standard deviation

Table 2 shows the adjusted association between mean annual temperature and binge drinking over the last year using any type of alcoholic beverage. We observed a non-significant increase in the prevalence of binge drinking for every increase of 1 °C across municipalities of the same region in all models.

**Table 2:** Associations of mean annual temperature with binge drinking in the last year among current drinkers

		Model 1^a^		Model 2^a^		Model 3^a^
	n	PR (95% CI)	n	PR (95% CI)	n	PR (95% CI)
Binge drinking of any alcoholic beverage						
*1 °C*	10,552	1.006 (0.997, 1.015)	6,893	1.009 (0.997, 1.021)	1981	1.013 (0.994, 1.033)

PR: prevalence ratio

Observation units are individuals nested within municipalities. The model fitted is a multilevel Poisson regression with a fixed effect for each region

Model 1 includes all the sample

Model 2 includes only individuals without temperature missing values

Model 3 includes only individuals without temperature missing values and within the first km from the weather station

^a^Adjusted for age, sex, education level, marital status, and socioeconomic status

Table 3 shows the adjusted association between mean annual temperature and binge drinking in the last year by type of alcoholic beverage. An increase of 1 °C in mean annual temperature across municipalities of the same region increased by 0.9% (PR = 1.009, 95%CI 1.005, 1.013) the prevalence of beer binge drinking in those who consumed beer in the last year. In the sensitivity analyses, we obtained the same point estimates, although with larger confidence intervals. Associations for other alcoholic beverages were not significant, except for coolers’ binge drinking in model 3. We observed that an increase of 1 °C in mean annual temperature across municipalities of the same region increased by 3.0% (PR = 1.030, 95%CI 1.003, 1.057) the prevalence of coolers’ binge drinking in those who consumed coolers in the last year.

**Table 3:** Mean annual temperature associated with binge drinking by type of alcohol in the last year in current drinkers

	Model 1^a^	Model 2^a^	Model 3^a^
	PR (95%CI)	PR (95%CI)	PR (95%CI)
Beer binge drinking	(*n* = 8,911)	(*n* = 5,897)	(*n* = 1,711)
*1 °C*	1.009 (1.005, 1.013)	1.008 (1.004, 1.013)	1.008 (0.997, 1.019)
Liquor binge drinking^b^	(*n* = 3,832)	(*n* = 2,398)	(*n* = 720)
*1 °C*	1.047 (0.994, 1.102)	1.057 (0.988, 1.31)	0.997 (0.978, 1.017)
Wine binge drinking^b^	(*n* = 1,823)	(*n* = 1,227)	(*n* = 377)
*1 °C*	1.047 (0.944, 1.161)	0.996 (0.879, 1.130)	1.006 (0.984, 1.029)
Coolers binge drinking^b^	(*n* = 1,348)	(*n* = 886)	(*n* = 294)
*1 °C*	0.978 (0.901, 1.063)	1.024 (0.920, 1.140)	1.030 (1.003, 1.057)

PR: prevalence ratio.

Observation units are individuals nested within municipalities. The model fitted is a multilevel Poisson regression with a fixed effect for each region.

Model 1 includes all the sample.

Model 2 includes only individuals without temperature missing values.

Model 3 includes only individuals without temperature missing values and within the first km from the weather station.

^a^Adjusted for age, sex, education level, marital status, and socioeconomic status.

^b^Cubic polynomial

## Discussion

We aimed to estimate the association of annual mean temperatures with binge drinking, overall, and by type of alcoholic beverage. We did not observe an association between annual mean temperature at the municipality level and binge drinking of any alcoholic beverage in the last year. By type of alcoholic beverage, we observed that people living in municipalities of the same region with higher mean temperature reported higher beer binge drinking in the last year. In the sensitivity analysis, the results had the same direction but lost significance, which can be a result of a decrease in power due to a smaller sample size. The post hoc power was 97% with a 5% alfa and 90% with a 1% alfa for the observed effect using the whole sample. We also observed that coolers’ binge drinking prevalence increased with temperature when restricting to temperature values of weather stations without missing values and within the first km of the municipality.

Prior studies have reported an association between temperature and binge drinking of any alcoholic beverage, although the direction of associations is mixed. In Scotland, the prevalence of binge drinking in the last month of any alcoholic beverage was higher in late autumn, but 10 or more episodes of binge drinking in the last month were higher during summer when temperatures are higher [13]. In the US, Ventura, et al., found a negative correlation (*R*=-0.6) between the prevalence of binge drinking of any alcoholic beverage and temperature across states [14]. Methodological differences may explain the heterogeneity of results. The study in Scotland collected data at an individual level and described monthly trends—not temperature— without adjusting for key variables that might confound the association (e.g., education level, socioeconomic status). The study in the US is ecological and the age-standardized prevalence of binge drinking was calculated for the whole population and not just among current drinkers. As previously suggested by other authors [15, 16], we measured binge drinking at an individual level, adjusted models for sex, education, and socioeconomic status, potential confounders at the individual level, and restricted comparisons to the same region, to control for sociocultural factors at the regional level, such as alcohol preference and perception. Also, we defined binge drinking as the consumption of 4/5 drinks per occasion, although the limitations of current definitions of binge drinking have been widely discussed [29]. The analysis of temperature and alcohol consumption faces significant methodological challenges that need to be discussed and clarified; further studies should strive to reduce potential confounding when estimating associations of binge drinking with temperature.

Another explanation for the mixed results between studies is that the relationship of temperature with an overall measure of alcohol use could lead to different results depending on the dominant alcoholic beverage consumed at the national, regional, and state levels. We found that beer binge drinking is associated with higher temperatures in current beer drinkers, but we did not observe similar behavior for other alcoholic beverages. This could be explained by the perception of beer as a refreshing cold beverage in the Americas [30], which is also encouraged by beer companies [31]. Yet, the cultural perception of alcohol is not homogeneous. For instance, in Germany wine is often consumed as a hot beverage during winter, consequently perceived as a “hot” beverage [32]. The association of binge drinking due to any type of alcohol will largely depend on the dominant beverage and the cultural framework upon which such beverage is consumed. This highlights the importance of considering the cultural context when studying alcohol consumption as an outcome of interest to better understand the implications of the findings.

The increase of 0.9% in the prevalence of beer binge drinking for each increase in 1 °C that we observed in our study might seem relatively small, yet it would represent around 196,000 more adults in this pattern of beer consumption for every degree of temperature. This estimation is considering that from 2007 to 2017 Mexico had an average increase of 1 °C [9], and that by 2016, 45.0% of adults 18 and older reported consuming beer in the last year, and of those, 73.1% reported at least one episode of binge drinking in the same time frame (*n* = 21.8 million). This has important implications because the probability of engaging in risky behaviors (e.g., drunk driving, alcohol poisoning) while consuming beer at hot temperatures could be higher because the perception of risk is lower for beer consumption compared to other alcoholic beverages [33]. These findings highlight the urgency of strengthening current policies and implementing new ones that address climate change, especially in hotter municipalities, to prevent multiple adverse health outcomes, including those by excessive alcohol consumption. At the same time, it is necessary to continue with the implementation of strategies that prevent and reduce alcohol use, such as the SAFER initiative [34] recommended by the WHO.

Some limitations of our study need to be acknowledged. First, participants usually underreport their alcohol consumption in surveys, which can drive the association toward the null, although this is not always the case [35]. Second, the prevalence of wine and coolers drinking among current drinkers is low (17.2% and 12.8%) so we might not be capturing their association with temperature because of their low prevalence. Third, when comparing the sociodemographic characteristics, we found more men [6% points (pp)], more with primary and middle-school education (6.1 and 5.9 pp), and less in the highest quarter of SES among those excluded versus those included (Additional Table 2). This could impact the generalizability of the results but not the internal validity of the estimates. Fourth, we are using mean annual temperature as exposure which might not be enough to capture the variability to which individuals are exposed throughout the year and we cannot ascertain the date of the binge drinking. This is a limitation of the time frame of the outcome measurement (binge drinking in the last year). Fifth, the establishment of causality between temperature and beer binge drinking is hard since the data are cross-sectional, and residual confounding is likely since there may be other characteristics across municipalities that could explain the association.

## Conclusions

Our results suggest that within the same region of the country, beer binge drinking was more likely in municipalities with higher temperatures. If this finding were to be confirmed by robust longitudinal studies, high ambient temperature, and temperature increases should be considered among important factors for alcohol control, as stronger interventions to prevent binge drinking could be needed in these areas. As temperature rises, mitigation and adaptation strategies to reduce exposure to high temperatures could also help reduce binge drinking and the alcohol-related burden of disease in Mexico.

### Electronic supplementary material

Below is the link to the electronic supplementary material.

**Table A.1**. Characteristics of current drinkers in the last year

**Table A.2**. Characteristics of individuals excluded due to missing information on temperature and individual included in the analysis.

**Additional Fig.1**. Prevalence of binge drinking in the last year and mean temperature in 1°C intervals in current drinkers.

**Additional Fig.2**. Prevalence of beer binge drinking in the last year and mean temperature in 1°C intervals in current drinkers of beer.

**Additional Fig.3**. Prevalence of liquor drinking in the last year and mean temperature in 1°C intervals in current drinkers of liquor.

**Additional Fig.4**. Prevalence of wine binge drinking in the last year and mean temperature in 1°C intervals in current drinkers of wine.

## Data Availability

The datasets analysed during the current study are available in the INSP repository, [https://encuestas.insp.mx/repositorio/encuestas/ENCODAT2016/] and the CONAGUA repository [https://smn.conagua.gob.mx/es/climatologia/informacion-climatologica/informacion-estadistica-climatologica].
