# Supplementary material for: Binge drinking associated with mean temperature: a cross-sectional study among Mexican adults living in cities
Source: Global Health. 2024 Apr 12;20:29. doi: 10.1186/s12992-024-01033-z (PMC11010420; doi:10.1186/s12992-024-01033-z)
Supplement: Supplementary file 1 — Table A.1. Characteristics of current drinkers in the last year [file 12992_2024_1033_MOESM1_ESM.docx]

|  | All sample | Sensitivity 1 | Sensitivity 2 |
| --- | --- | --- | --- |
|  | n (%) | n (%) | n (%) |
| Age, years (mean, ± SD) | 37.5 ± 12.8 | 37.5 ± 12.8 | 37.9 ± 12.8 |
| Sex |  |  |  |
| Male | 5,392 (51.1) | 3,481 (50.5) | 3,557 (51.6) |
| Female | 5,160 (48.9) | 3,412 (49.5) | 3,336 (48.4) |
| Education |  |  |  |
| None | 549 (5.2) | 358 (5.2) | 345 (5.0) |
| Elementary | 1,541 (14.6) | 1,013 (14.7) | 931 (13.5) |
| Middle-school | 3,651 (34.6) | 2,413 (35.0) | 2,226 (32.3) |
| High-school | 3,028 (28.7) | 1,930 (28.0) | 2,006 (29.1) |
| Graduate | 1,783 (16.9) | 1,172 (17.0) | 1,379 (20.0) |
| Marital status |  |  |  |
| Single | 3,081 (29.2) | 2,020 (29.3) | 2,047 (29.7) |
| Cohabitating | 6,257 (59.3) | 4,067 (59.0) | 4,012 (58.2) |
| Separated/Divorced | 992 (9.4) | 662 (9.6) | 6,82 (9.9) |
| Widowed | 222 (2.1) | 145 (2.1) | 145 (2.1) |
| Socioeconomic status |  |  |  |
| Q1 | 1,414 (13.4) | 841 (12.2) | 834 (12.1) |
| Q2 | 1,889 (17.9) | 1,220 (17.7) | 1,144 (16.6) |
| Q3 | 2,912 (27.6) | 1,930 (28.0) | 1,792 (26.0) |
| Q4 | 4,337 (41.1) | 2,902 (42.1) | 3,129 (45.4) |

Table A.1. Characteristics of current drinkers in the last year

SD: standard deviation
