# Supplementary material for: Binge drinking associated with mean temperature: a cross-sectional study among Mexican adults living in cities
Source: Global Health. 2024 Apr 12;20:29. doi: 10.1186/s12992-024-01033-z (PMC11010420; doi:10.1186/s12992-024-01033-z)
Supplement: Supplementary file 2 — Table A.2. Characteristics of individuals excluded due to missing information on temperature and individual included in the analysis. [file 12992_2024_1033_MOESM2_ESM.docx]

|  | Excluded | Included |  |
| --- | --- | --- | --- |
|  | n (%) | n (%) | P value |
| Age, years (mean, ± SD) | 37.4 ± 12.3 | 37.5 ± 12.8 | 0.9^a^ |
| Sex |  |  | 0.01^b^ |
| Male | 350 (56.0) | 5,802 (50.8) |  |
| Female | 275 (44.0) | 5,432 (49.2) |  |
| Education |  |  | <0.001 ^b^ |
| None | 28 (4.5) | 575 (5.2) |  |
| Elementary | 130 (20.8) | 1,624 (14.7) |  |
| Middle-school | 254 (40.6) | 3,834 (34.7) |  |
| High-school | 139 (22.2) | 3,161 (28.6) |  |
| Graduate | 74 (11.8) | 1,840 (16.7) |  |
| Marital status |  |  | 0.4 ^b^ |
| Single | 164 (26.2) | 3,229 (29.3) |  |
| Cohabitating | 384 (61.4) | 6,539 (59.3) |  |
| Separated/Divorced | 64 (10.2) | 1,032 (9.4) |  |
| Widowed | 13 (2.1) | 234 (2.1) |  |
| Socioeconomic status |  |  | <0.01 ^b^ |
| Q1 | 80 (12.8) | 1,498 (13.6) |  |
| Q2 | 127 (20.3) | 1,978 (17.9) |  |
| Q3 | 203 (32.5) | 3,041 (27.6) |  |
| Q4 | 215 (34.3) | 4,517 (40.9) |  |

Table A.2. Characteristics of individuals excluded due to missing information on temperature and individual included in the analysis.

SD: standard deviation

^a^T-test

^b^Chi-squared test.
