# Supplementary figures and images for: Binge drinking associated with mean temperature: a cross-sectional study among Mexican adults living in cities
Source: Global Health. 2024 Apr 12;20:29. doi: 10.1186/s12992-024-01033-z (PMC11010420; doi:10.1186/s12992-024-01033-z)

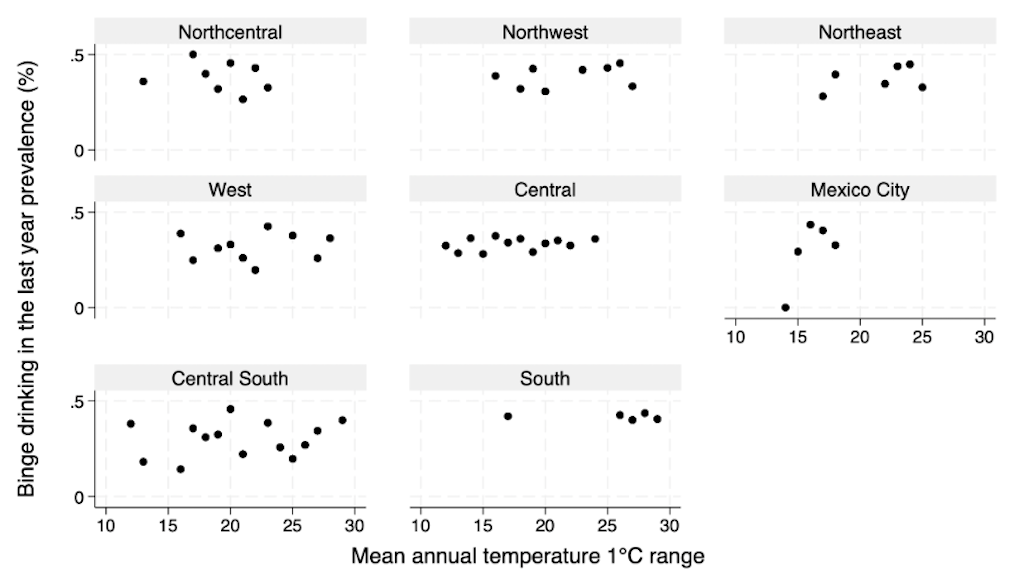

Supplement: Supplementary file 3 — Additional Fig.1. Prevalence of binge drinking in the last year and mean temperature in 1°C intervals in current drinkers. [file 12992_2024_1033_MOESM3_ESM.png]

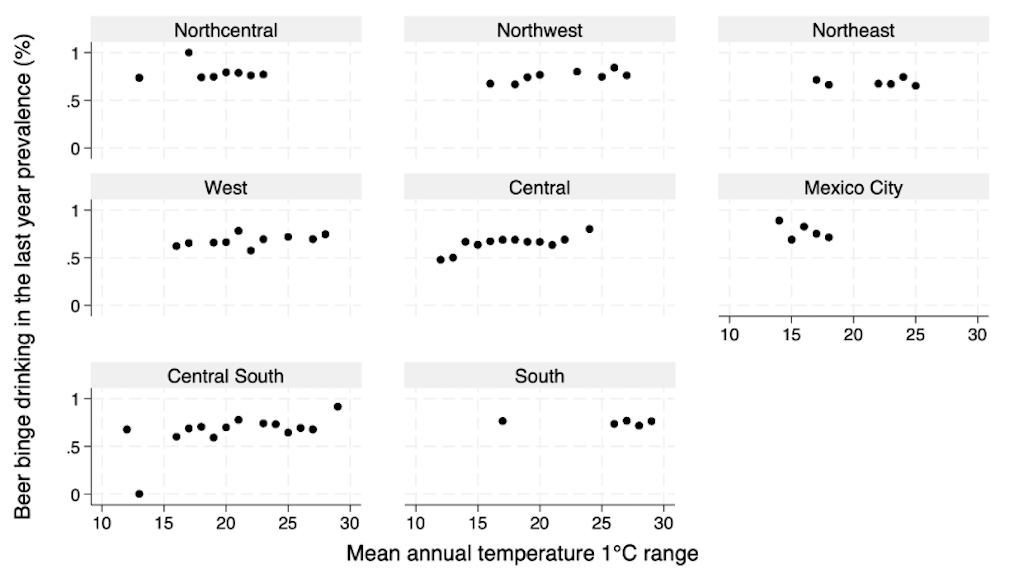

Supplement: Supplementary file 4 — Additional Fig.2. Prevalence of beer binge drinking in the last year and mean temperature in 1°C intervals in current drinkers of beer. [file 12992_2024_1033_MOESM4_ESM.png]

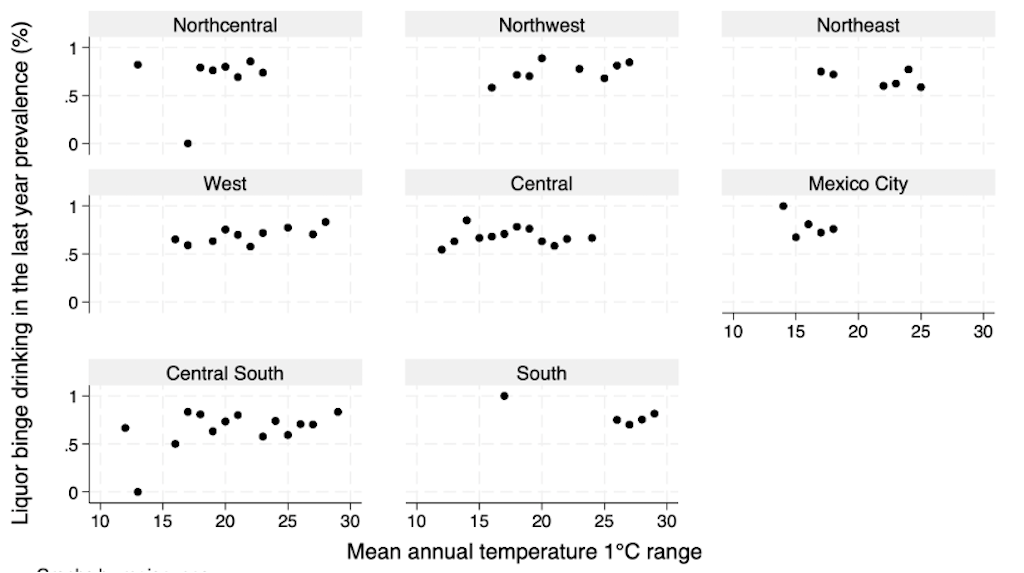

Supplement: Supplementary file 5 — Additional Fig.3. Prevalence of liquor drinking in the last year and mean temperature in 1°C intervals in current drinkers of liquor. [file 12992_2024_1033_MOESM5_ESM.png]

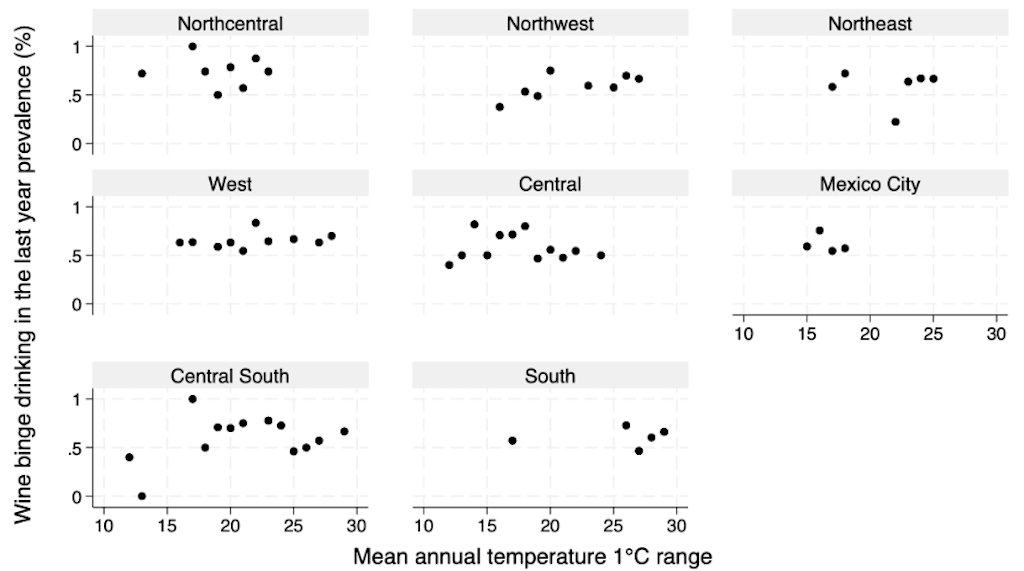

Supplement: Supplementary file 6 — Additional Fig.4. Prevalence of wine binge drinking in the last year and mean temperature in 1°C intervals in current drinkers of wine. [file 12992_2024_1033_MOESM6_ESM.png]
